# A Danish version of the life-space assessment (LSA-DK) – translation, content validity and cultural adaptation using cognitive interviewing in older mobility limited adults

**DOI:** 10.1186/s12877-019-1347-0

**Published:** 2019-11-15

**Authors:** Mette Merete Pedersen, Pia Kjær-Sørensen, Julie Midtgaard, Cynthia J. Brown, Ann Christine Bodilsen

**Affiliations:** 1 Clinical Research Centre, Copenhagen University Hospital Hvidovre, Kettegård Alle 30, 2650 Hvidovre, Denmark; 2 Physical Medicine Research-Copenhagen (PMR-C), Copenhagen University Hospital Hvidovre, Hvidovre, Denmark; 3 Faculty of Health and Medical Sciences, University of Copenhagen, Copenhagen, Denmark; 4 The University Hospitals’ Centre for Health Research, Copenhagen University Hospital Rigshospitalet, Copenhagen, Denmark; 5 Department of Public Health, Faculty of Health and Medical Sciences, University of Copenhagen, Copenhagen, Denmark; 6 Birmingham/Atlanta Geriatric Research, Education and Clinical Center, University of Alabama at Birmingham, Birmingham, AL USA; 7 Exercise and Health, Roskilde Municipality, Roskilde, Denmark

**Keywords:** Life-space assessment, Older adults, Cognitive interviewing, Mobility

## Abstract

**Background:**

Identification and prevention of mobility limitations in older adults is important to reduce adverse health outcomes. The Life-Space Assessment (LSA) provides a single measure of mobility including environmental and social resources of the older adult. Availability of the LSA for non-English speaking countries is still sparse. Therefore, we translated the LSA into Danish and performed a content validity analysis of the translation in older adults with mobility limitations.

**Methods:**

After translation into Danish, the Danish version (LSA-DK) was content validated using cognitive interviewing in older mobility limited adults (+ 65) from an outpatient rehabilitation center (*n* = 12), medical wards at a university hospital (*n* = 11), and an assisted living facility (*n* = 7). The interviews were transcribed and analyzed according to the four stages of the Information Processing Model. Based on the analyses, recommendations for changes to the LSA-DK and to the manual were made and presented to the developers of the LSA.

**Results:**

Consensus was reached on the LSA-DK. Thirty cognitive interviews were carried out. A wide range of sources of error primarily related to the comprehension, memory and decision process were identified. The frequency and type of error sources were most prevalent among assisted living facility informants and included difficulties in defining the geographical extension of neighborhood, town and outside town. The results led to adaptations to the questionnaire and manual to support implementation of the LSA-DK in clinical practice.

**Conclusions:**

The Life-Space Assessment was translated into Danish and content validated based on cognitive interviews. Adaptations were made to support that the Danish version can be implemented in clinical practice and used in the assessment of mobility in older Danish adults.

## Background

Mobility is a central component of healthy aging [1, 2]. According to Satariano et al. mobility refers to *“movement in all of its forms, including basic ambulation, transferring from a bed to a chair, walking for leisure and the completion of daily tasks, engaging in activities associated with work and play, exercising, driving a car, and using various forms of public transport*” [1]. Hence, mobility is closely related to maintenance of autonomy and independent living in older adults. Accordingly, mobility limitations lead to constricted life space and may have serious consequences for older adults and their ability to sustain physical capacity, social relationships, and quality of life [1, 3, 4]. Therefore, arguments support early identification of mobility limitations in older adults as mobility limitations are associated with significant adverse health outcomes [2, 5–8]. Several mobility assessment instruments are available for use in older adults including accelerometers [9, 10], various physical performance tests and self-reported questionnaires [11–15]. Common for these measures is that they assess mobility on the activity level of the International Classification of Functioning, Disability and Health (ICF) [16]. Measures that can evaluate older adults’ dependency in mobility, how far and how often they are moving in the surrounding environment are generally sparse.

The University of Alabama at Birmingham (UAB) Study of Aging Life-Space Assessment (LSA) tool was developed for community-dwelling older adults (+ 65 years) [17]. The LSA provides a single measure of mobility and change in mobility at the participation level of the ICF [16], encompassing environmental and social resources of the older adult [17]. The LSA has demonstrated high test-retest reliability using phone interviews over at 2-week period [17], validity [18] and sensitivity to change [17], and correlates well with physical activity in community-dwelling older adults [19]. Also, the LSA has been translated into different languages and has shown acceptable reliability and validity in these settings [20–24]. However, the LSA has not yet been translated into Danish and content validated for use in older, mobility limited Danish adults.

Therefore, the aim of this study was three-fold: 1) to translate the Life-Space Assessment into Danish, 2) to validate the content of the Danish version by means of cognitive interviewing in a population of both community-dwelling and hospitalized older adults as well as assisted living facility residents, to reflect different degrees of mobility limitations, and 3) to culturally adapt the questionnaire according to the findings of the content validity analysis.

## Methods

The study consists of three phases: 1) a translation/back-translation process, 2) an evaluation of the translated version with respect to content validity, and 3) a cultural adaptation process in which the questionnaire was modified according to the findings of the content validity analysis.

The study was approved by the Danish Data Protection Agency (No.: 11-11-2016) and the local Ethics Committee of the Capital Region (No.: H-16050540). Informed written consent was obtained from all informants after provision of both oral and written information in accordance with The Declaration of Helsinki. The study follows the Qualres Guidelines for qualitative research [25].

### The life-space assessment (LSA)

The LSA [17] is an interviewer-based questionnaire assessing information on the distance a person reports moving during the 4 weeks leading up to the assessment. Information is collected about movement from the room where the person sleeps to five consecutive life-space levels (LS1-LS5): 1) within the home; 2) outside the home; 3) in the neighborhood, 4) outside the neighborhood, 5) and outside the town (Table 1). For each of the five levels, informants are asked if the level was attained over the last 4 weeks (yes/no), at what weekly frequency (less than once a week, 1–3 times per week, 4–6 times per week, daily) and whether equipment such as canes or walkers was needed (yes/no) or if help from another person was needed (yes/no). A composite score (0–120 points) is calculated based on the life-space level attained, the frequency of attainment and the degree of independence. A manual describing testing and scoring procedures can be obtained from the author (pbaker@uab.edu). A non-validated Danish translation of the manual was produced in the process of translating the LSA. The contents of the manual was approved by the developers of the LSA. The manual is available from the corresponding author of this paper or at Zenodo.org (see the data availability statement).

**Table 1:** Levels of the Life Space Assessment

LSA level	During the last four weeks have you been to…	At what weekly frequency?	Was equipment such as a cane or walker needed?	Was assistance from a person needed?
Level 1	…other rooms of your home besides the room where you sleep?	1) less than once a week 2) 1–3 times per week 3) 4–6 times per week, 4) daily	1) Yes 2) No	1) Yes 2) No
Level 2	…an area outside your home?	1) less than once a week 2) 1–3 times per week 3) 4–6 times per week, 4) daily	1) Yes 2) No	1) Yes 2) No
Level 3	…places in your neighborhood?	1) less than once a week 2) 1–3 times per week 3) 4–6 times per week, 4) daily	1) Yes 2) No	1) Yes 2) No
Level 4	…places outside your neighborhood?	1) less than once a week 2) 1–3 times per week 3) 4–6 times per week, 4) daily	1) Yes 2) No	1) Yes 2) No
Level 5	…places outside your town?	1) less than once a week 2) 1–3 times per week 3) 4–6 times per week, 4) daily	1) Yes 2) No	1) Yes 2) No

*LSA: Life Space Assessment*

### Translation of the LSA into Danish

We used a translation procedure following the recommendations put forward by the International Society for Pharmacoeconomics and Outcomes Research (ISPOR) [26].

1. Preparation: Written permission to translate the LSA was obtained from the instrument developers.
2. Forward Translation: Three native Danish (target language) speakers, fluent in English (source language), translated the LSA into the target language resulting in three independent translations (T1, T2 and T3). Two of the translators were familiar with the LSA and the LSA was unknown to the third translator.
3. Reconciliation: in a consensus meeting, the three translators compared and merged their translations (T1, T2 and T3) into a single common forward translation (T4).
4. Back Translation: The common version (T4) was back translated from the target language to the source language (B1) by a translator who was blind to the original version of the LSA.
5. Back Translation Review and harmonization: The back translated version (B1) and the original version were compared by the developers of the LSA to identify differences between the two versions resulting in a minor revision (B2) of the back translated version, which was approved by the instrument developers (B2A).
6. Cognitive Debriefing and review of Cognitive Debriefing results: The accepted version (B2A) was validated using cognitive interviewing (see below for a detailed description).
7. The final version was approved by the developers of the instrument and was checked for spelling and grammar issues and proofread after the lay-out was finalized (LSA-DK).

### Cognitive interviewing

Cognitive interviewing (CI) was used in the content validation of the LSA to identify problems and potential errors when administering the LSA-DK [27]. Cognitive interviewing is a widely acknowledged method in the development, validation and cultural adaptation of patient-reported outcome measures [27–29]. Cognitive interviewing may be used in parallel to translation and pretesting of questionnaires to help identify and thereby minimize the risk of systematic measurement bias [30]. Cognitive interviewing is useful when in doubt about the informants’ understanding of the wording of a question or how informants will interpret and answer questionnaires [28]. Also, it has proven to be a robust technique for the identification of response errors [31]. The cognitive interviews were based on an interview guide developed in accordance with Willis [28] and carried out through semi-structured interviews by a female occupational therapist (PKS), who was familiar with cognitive interviewing. The informants were asked to think aloud as they went through the LSA questions and to tell the interviewer everything they were thinking. The interviewer regularly asked probing questions to understand the informant’s thought process by using anticipated and conditional probes [28]. The interviews were audio-recorded and had a duration of approximately 30 min. The cognitive interviews were based on a priori process with the Question Appraisal System (QAS-99) where potential problems and limitations was identified [32]. The cognitive interviews were subsequently based on the principles of The Information Processing Model (IPM) developed by Tourangeau, which covers four cognitive stages: 1) Comprehension of the question; 2) Retrieval from memory of information necessary to answer the question; 3) Decision processes, relating to the adequacy of the answer; and 4) the Response process, where the informant produces an answer that satisfies the task requirements [33]. Based on a review of the four cognitive stages, we determined how the informant understood and responded to the questions and if the questions generated the information as intended.

### Informants

According to Beaton et al. [34] a sample size of 30–40 informants was needed. We used criteria-based sampling aiming at maximum variation to establish informant variability according to gender, age, educational level, mobility and cognition [27, 35]. Hence, informants were recruited among older people in the Copenhagen area and enrolled from: 1) an outpatient rehabilitation center; 2) medical wards at a university hospital, and 3) an assisted living facility. The inclusion criterion was: 55 years or older. The exclusion criteria were: inability to speak Danish; inability to cooperate in a cognitive interview; and inability to participate in interviewing due to speech difficulties. The cognitive interviews were performed either at the hospital ward (for those hospitalized) or in the informant’s home. Demographic variables were collected based on self-report in connection with the interviews and encompassed educational level, marital status, comorbidities, cognitive function by the Orientation Memory Concentration test (OMC) [36], and mobility by the New Mobility Score (NMS) [37].

### Data analysis

The interviews were transcribed and analyzed inspired by the *template organizing style* as described by Crabtree [38]. From this analysis, content codes were sorted according to the four stages of the IPM [33] and categorized in a systematic matrix-contruction [39]. The results of the analysis were discussed by three authors until consensus was reached on recommendations for changes to the questionnaire and to the manual. These recommendations were presented to the developers of the LSA before any changes were made.

## Results

### Translation

During the translation process, minor discrepancies were identified between the three translators, who agreed on a consensus version of the translation before this agreed upon version was back-translated into the source language. During the harmonization process, the developers accepted the use of “during the last 4 weeks” instead of “during the last month” in the introduction sentence of the questionnaire as well as the use of one Danish word covering the words “aids and equipment”. Apart from this, the translated version was identical to the original version with regards to content and wording.

**Fig. 1:**
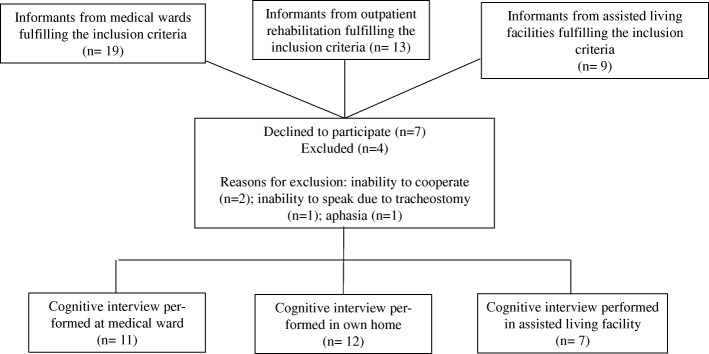
Flow of informants. Flow of informants from a medical ward, an outpatient rehabilitation center and an assisted living facility

Table 2 describes the characteristics of the sample participating in the cognitive interviews. The flow of informants can be seen in Fig. 1. A total of 41 potential informants living in municipalities around the city of Copenhagen were given written and oral information. Eleven declined participation and four were excluded – two were unable to cooperate, one could not speak due to a new tracheostomy, and one had aphasia. A total of 30 cognitive interviews were carried out in March 2017. The final sample consisted of 12 community-dwelling informants, 11 hospitalized informants and 7 assisted living facility residents. The 7 assisted living facility informants had a lower median cognitive level (OMC 18.7, range 16–23) than the community-dwelling informants (OMC 25.8, range 18–28) and those who were hospitalized at the time of the assessment (OMC 24.5, range 14–28) (results not shown).

**Table 2:** Informant characteristics

	All informants *N* = 30	Informants in own home *N* = 12	Hospitalized informants *N* = 11	Assisted Living Facility informants *N* = 7
Age, mean (range)	78.7 (60–97)	82.4 (73–90)	71.8 (60–92)	83.3 (71–97)
Sex (female), N (%)	15 (50.0)	7 (58.3)	5 (45.5)	3 (42.9)
Married, N (%)	8 (26.7)	3 (25.0)	5 (45.5)	**–**
Type of residence, N (%)				
Apartment	26 (86.7)	12 (100)	7 (63,6)	7 (100)
House	4 (13.3)	–	4 (36.4)	–
Education				
≤ 10 years, N (%)	5 (16.7)	2 (16.6)	1 (9.1)	2 (28.6)
10–15 years, N (%)	12 (40.0)	5 (41.7)	4 (36.4)	3 (42.9)
> 15 years, N (%)	13 (43.3)	5 (41.7)	6 (54.5)	2 (28.6)
Comorbidities, number (range)	1.3 (0–3)	1.0 (0–3)	1.9 (0–3)	1.0 (0–2)
OMC, (0–28 point), mean (range)	23.7 (14–28)	25.8 (18–28)	24.5 (14–28)	18.7 (16–23)
NMS, (0–9 point), mean (range)	6.6 (3–9)	8.8 (3–9)	7.6 (3–9)	5 (4–7)

*OMC Orientation Memory Concentration test, NMS New Mobility Score.*

### Cognitive interviews

Overall, the Danish version of the LSA questionnaire was well accepted by the informants. During the cognitive interviews, almost half of the informants displayed challenges concerning each of the five levels of the LSA: 60.0% for LS1, 73.3% for LS2, 46.7% for LS3, 83.3% for LS4 and 50.0% for LS5. The challenges concerned one or more of the four levels of the IPM: IPM1) comprehension, IPM2) retrieval from memory, IPM3) decision process, or IPM4) response process. However, most of the discrepancies were related to IPM1 and IPM2, i.e. comprehension of the questions and retrieval from memory.

### IPM1: comprehension of the question

During the cognitive interviews, several challenges appeared concerning comprehension of the questions of the LSA. One third of the informants expressed difficulties comprehending the contents of the word “activities” in the introductory sentence “these questions refer to your activities just within the past four weeks”, the meaning of which was understood as e.g. physical exercise, strength training or outdoor activities. Also, challenges comprehending some of the essential words used in the LSA levels emerged. Some informants expressed doubt about the meaning of “been to”:

*What do you mean by been to? If I have passed by? Or if I have spent time there? How should it be understood? (community-dwelling informant)*

Half of the informants (15/30) needed clarifications about the LS1 question: ‘Have you been to rooms other than the room where you sleep?’ (3/12 community-dwelling; 5/11 from hospital; and 7/7 assisted living facility residents). Informants living in one-bedroom apartments had the greatest difficulty comprehending the question.

For LS3-LS5, one to two thirds of the informants (11/30 for LS3, 20/30 for LS4 and 12/30 for LS5) had difficulties comprehending the question, e.g. difficulties defining neighborhood, town and outside town. Relatively more assisted living facility residents than informants from own home and hospitalized informants expressed difficulties. The difficulties concerned defining the geographical extension of neighborhood and town. For some informants, neighborhood was understood as being something relational (neighbors, family etc.) and not geographical:

*My neighborhood is my son in the next-door quarter of town (community-dwelling informant).*

Also, some informants failed to include all their community mobility in their answers. For example, some informants did not consider going to a rehabilitation session as being community mobility that would be included in the response.

*Yes, (I did not consider rehabilitation)… as I thought about my family and such… but that’s right… (community-dwelling informant*

In addition, the frequency item was understood and answered by some informants as describing the preceding month and not being a weekly average. In connection to items on equipment and personal assistance, some informants not able to drive a car themselves did not consider a driver to be “help from another person” and some did not consider furniture used for balance as being equipment:

*Sometimes, I get up at night, when it’s dark, and use the wall for support. But you can’t really call that an equipment (community-dwelling informant).*

Also, variation in the understanding of when one can be considered using equipment was seen:

*I don’t use any of it (…). I only use the walking stick as a precaution, if I go out and if there’s a storm, because my balance is a bit out of control (community-dwelling informant).*

### IPM2: retrieval from memory of information necessary to answer the question

The informants displayed challenges remembering where they had been to in the last 4 weeks. Also, for two thirds of the informants (19/30), the contents of LS2 seemed complex and the informants could not remember the entire question. After hearing the LS2 question, an informant answered:

*Oh boy. That was a lot. If I’ve been outside the area, my porch, garage… that was a lot of things (assisted living facility informant).*

In addition, some informants were challenged when answering the frequency questions since they forgot which time frame they had to consider (4 weeks).

### IMP3: decision processes relating to the adequacy of the answer

Challenges regarding the decision process were related to lack of attention and concentration affecting the adequacy of the answers.

*Yes. I think they (the items) overlapped ... (…). Where are you now… oh, you’re at home, no you’re not at home, now you’re here. It was a bit difficult for me (…). Or maybe it’s because I’m tired… (hospitalized informant).*

When asked how they decided on a specific answer, the informants answered:

*It’s difficult… and I cannot promise you that it’s correct (…). It’s not something that I think about, I guess (hospitalized informant)*

Also, some informants considered a trip to the supermarket to be a frequency of 2, thus counting both ways.

### IMP4: response process, where the informant produces an answer that satisfies the task requirements

Only a few informants expressed difficulties regarding response categories and how to respond to the questions. The observed challenges were seen in informants who were hospitalized and informants who had recently moved into an assisted living facility. Hospitalized informants, who had been hospitalized for more than a couple of days, found it difficult to understand which 4 weeks they had to consider in their answers and whether they had to include the time spent at the hospital in their answers. In relation to questions regarding independence (i.e. if help from another person was needed), both hospitalized informants and assisted living facility informants tended to consider two locations (home vs. hospital/assisted living facility) when answering. For these two categories of informants, the fact that personal assistance was available 24 h a day (from staff) complicated their answers.

*No, it… yes, well I have the staff. They are here all day (…) (assisted living facility informant).*

### Challenges with the cognitive interviewing method

In general, informants from assisted living facilities displayed more challenges with the cognitive interviewing method than informants living in own home or hospitalized informants – challenges were observed in 85.7% assisted living facility residents, 54.4% hospitalized informants and 33.3% of community-dwelling informants.

### Recommendations for revision of questionnaire and manual

The analysis of the cognitive interviews resulted in recommendations for a cultural adaptation consisting of changes and additions, which are presented in Table 3. Two of these recommendations led to revision of the questionnaire: a parenthesis was added to LS1 and two examples were removed from LS3. The remaining recommendations, some of which were context dependent, encompassed explanations and probes added to the manual to facilitate informant comprehension of the questionnaire and guide interviewers in how to use the questionnaire. For example, it was added that for respondents in hospital wards the interviewer should stress that the questions refer to the 4 weeks preceding hospital admission.

**Table 3:** Changes to the Danish Life Space Assessment (LSA-DK) following cognitive interviews

IPM Stages	*Challenges discovered in cognitive interviews and actions taken*	*Changes made to the LSA-DK or to the Danish LSA manual*
*Comprehension*	Problems understanding the word “activities”. To avoid misunderstandings regarding the focus of the LSA, an introductory sentence was added to the Danish manual.	Added to manual *“The questions I am about to ask you, cover how far, how often and how you have gotten around during the last four weeks”*
	Problems understanding the phrase “been to”. A probe was added to the Danish manual.	Added to manual *“It doesn’t matter for how long you’ve been in a room/an area. I just need to know if you’ve been in the room/the area or if you’ve passed through the area”*
	In LS1, “other rooms of your home…” was often not comprehended by respondents living in one-bedroom apartments. Changes were made in both the LSA-DK and the Danish manual.	Added to questionnaire A parenthesis with examples was added to the LS1 question. *“Other rooms of your home besides the room where you sleep (*e.g. *the kitchen or the bathroom)”* Added to manual An explanatory text was added to the manual: “*The example in the parenthesis can be used to clarify ambiguities to the respondent. For example it would be appropriate to say*” *for example your kitchen or your bathroom” in Level 1 for respondents living in one-bedroom apartments”*
	The frequency item was understood and answered by some respondents as describing the preceding month and not being a weekly average. An explanatory note was added to the Danish manual.	Added to manual *“For frequency, it is important to verify the respondent’s answer to ensure that the frequency noted is an average over the last 4 weeks, representing frequency per week and not per month”*
	In connection with items on equipment, the respondents did not consider furniture used for balance or a walking stick used as a safety precaution as being equipment. The LSA manual already describes equipment used for safety. A probe was added to the Danish manual regarding furniture support.	Added to manual *“Do you support yourself using for example furniture, when walking around inside or outside?”*
	At LS3 and LS4, the spatial definitions of neighborhood and town caused misunderstandings among some respondents. No changes were made since no other Danish words are more suitable. The LSA manual’s definitions of neighborhood and town are recommended to be used to clarify the questions.	
*Retrieval from memory*	In LS2, problems remembering the content due to the length of the question were identified. Some of the examples (patio and garage), representing synonyms, were extracted from the LSA-DK after permission from the developers.	New wording in questionnaire: *“An area outside your home such as your porch, deck, yard, hallway (of an apartment building) or your driveway?”*
*Decision processes and Response process*	Some respondents calculated the frequency to include both the trip to and the return trip from e.g. the supermarket. An explanatory text was added to the Danish manual. Some respondents were challenged when answering the frequency questions since they forgot which time frame they had to consider (4 weeks). A note was added to the manual.	Added to manual *“The interviewer must be aware, that some respondents will consider a trip to the supermarket to be a frequency of 2, thus counting both ways. This, however, should only be counted as 1, that is 1 time back and forth”.* Added to manual *“The interviewer must be aware of the necessity of using probes to elucidate responses regarding aids, personal assistance and frequency”.*

*IPM: The Information Processing Model*

## Discussion

During the translation process of the LSA, minor changes were made to the LSA-DK before the questionnaire was validated using cognitive interviewing. Overall, the LSA-DK was well accepted by the informants. During the content validity examination, we identified several potential challenges primarily regarding comprehension of the questions in LS1 and LS3 and memory issues regarding LS2. Two of the challenges led to minor changes of the questionnaire while others led to the addition of probes and explanations in the manual.

The consensus-based standards for the selection of health measurement instruments (COSMIN) initiative recommends evaluating the content validity of an outcome measurement instrument before evaluating other measurement properties, as lack of content validity can affect all other measurement properties [40]. Nevertheless, studies evaluating the content validity of translated LSA versions are still sparse. To our knowledge, only two studies have evaluated the content validity of translated versions of the LSA [20, 41]. Similar to our study, Auger et al. [20] used cognitive interviewing methods to evaluate the questionnaire. Consistent with Auger et al. [20], we found that a substantial proportion of informants needed clarifications regarding LS1: *‘Have you been to rooms other than the room where you sleep?’*. This challenge was overcome by adding “e.g. your kitchen or bathroom” in the LSA-DK questionnaire. Informants living in one-bedroom apartments had the greatest difficulty comprehending this particular question. Also, the analyses in both our study and the study by Auger et al. revealed that many informants had difficulty defining the geographical extension of neighborhood, town and outside town. Although several informants asked for a definition of both neighborhood and town, no changes were made based on these findings. Firstly, because no better words exist describing these two notions, and secondly since spatial definitions are provided in the manual for clarification if needed. Therefore, we recommend to carefully use and follow the manual when administering the LSA. Siordia has questioned the lack of defined geographical space in the questionnaire (e.g. neighborhood) since the informants define the geographical limits of neighborhood differently [42]. The LSA manual stresses the importance of letting the informants define for themselves to ensure that their definition of neighborhood does not change at repeated assessments, thus enabling evaluation of change over time for the individual. However, as stated by Siorda [42] there may be a risk of comparisons based on unequal grounds when comparing between-people differences, i.e. populations across different geographical areas, if there’s a sizable fluctuation in the geographical size of neighborhood or town. Therefore, in a future study it could be relevant to investigate cultural differences in the definition of neighborhood and town and the consequence of defining neighborhood and town for the informants.

Overall, two thirds of the informants in our study found the content of LS2 complex and difficult to retain. This is well in line with a study investigating readability scores of the LSA, which found that all LSA items were easily understandable except for LS2 [42]. To ease the understandability of LS2, some of the examples (patio and garage) were extracted from the LSA-DK questionnaire after permission from the developers.

In this study, informants displayed challenges remembering where they had been to in the last 4 weeks. Likewise, a previous study has shown older adults (+ 65) to have difficulties in recalling physical activity within the previous 7 days resulting in errors consisting of both under and over reporting [43]. Also, older adults remember less well than younger adults [44], thus one would expect a greater risk of recall bias in the older adult population. However, the LSA has been shown to correlate well with a range of mobility measures, both objective and subjective, supporting the use of the LSA as a valid measure of mobility in older adults [19, 45]. A study evaluating the concurrent validity of the Swedish version of the LSA in 312 community-dwelling older adults (+ 75 years) found the LSA to correlate with mobility ability as measured by other mobility related measures (the Short Physical Performance Battery; stair climbing; transfer; transportation; food shopping; travel for pleasure; community activities) [46]. Also, a Finish study in community-dwelling older adults found Life-Space mobility to be correlated with objectively measured physical activity, i.e. step counts and activity time [19]. Similarly, a future step in the evaluation of the LSA-DK will be to investigate other measurement properties, i.e. criterion validity, reliability and responsiveness [40].

The LSA uses a *stem and leaf* design that has been shown to create confusion in older adults regarding determining responses when used in structured questionnaires [47]. However, a study in older Latino Americans (+ 80) found that response patterns in the LSA were not affected by the *stem and leaf* format and that informants did not respond illogically and falsely [42]. This difference may be due to the interview based character of the LSA [18] as to why the *stem and leaf* format is not expected to cause confusion in the LSA. This, however, requires that the interviewer is trained in and familiar with the manual and the questionnaire before conducting the interview.

### Strengths and limitations

In this study, we have provided a transparent translation of a mobility instrument which is widely used to assess mobility on the participation level of the ICF [16, 48–54]. This translation and translations of the instrument into other languages can facilitate comparison of mobility status across countries and nationalities. The main strength of the study is the inclusion of a diverse group of mobility limited older adults. In cognitive interviews, the informant characteristics must be similar to the target population [27]. Therefore, we chose a sample of older adults with different degrees of mobility limitations, since this reflects the population likely to be participating in clinical trials where the LSA-DK will be used. We strived for a sample of informants represented by sufficient information power [55] with regard to age, sex, place of residence, education, cognition and mobility. We chose to include assisted living facility residents, although the LSA was not originally developed for this population. The reason for this choice was that many Danish assisted living facility residents are cognitively well functioning and have community mobility despite living in an assisted living facility. However, even though this study is based on few assisted living facility informants this group of informants showed more difficulties with the questionnaire than independent living or hospitalized informants. Thus, using the LSA in assisted living facility residents is not recommended.

There is no consensus in the literature about the number of informants used in cognitive interviews [35]. Beaton et al. [56] recommend the use of 30–40 informants. In the present study 30 informants were included. Though we achieved a variety of informants with regards to age, sex, mobility level and education it cannot be ruled out that our sample size was too small to reach informational redundancy [35]. According to Blair et al. [57] it is far from certain that all problems are detected in samples over 30 and that even 50 might be too few. Thus, it is likely that this study has identified a broad variation of challenges, but some nuances may have been missed. Also, we could have wished for more informants from rural areas and more informants with less than 10 years of education, since this is the educational level for 1/4 of the Danish population over the age of 65 years [58].

Cognitive interviewing has been criticized for being too subjective [59]. However, we tried to overcome this criticism by using a standardized, systematic procedure when analyzing data [39]. In cognitive interviews, the interviewer plays a central role and needs competences in supporting *think aloud* and using appropriate *verbal probes* [35]. Some of the respondents needed support in differentiating between probes asked as part of the cognitive interview and probes related to the clarification of the different levels in LSA. Thus, the interviewer may have introduced bias into the data collection process. However, we have tried to reduce this risk by using an interview guide to standardize the use of probes [35]. Also, Wright and Holliday [60] found that cognitive interviewing as compared to structured interviews benefits the recall of older adults with and without mild cognitive impairment, suggesting that the use of cognitive interviewing may support the memory of older adults.

Finally, although the LSA manual provides a thorough explanation of how to administer the questionnaire and how to probe, it cannot be ruled out that errors related to the people administering the LSA can occur.

## Conclusions

In conclusion, the Life-Space Assessment was translated into Danish and content validated based on cognitive interviews. Adaptations were made to support that the translated version (LSA-DK) can be used in the assessment of mobility in older Danish adults. Before implementing the LSA-DK in clinical practice, however, further studies should be performed to investigate the reliability as well as the criterion validity of the LSA-DK.

## Data Availability

The data contain potentially identifying or sensitive information that could compromise the privacy of the respondents and are therefore not publicly available according to regulations set out by the Danish Data Protection Agency. The Danish version of the LSA (LSA-DK) as well as the manual are available at Zenodo.org (https://zenodo.org/record/3333418#.XShBArz_y9I (DOI: 10.5281/zonedo.3333418)).

## Abbreviations

B1: Back translation no 1 of T4
B2: Revised B1 after comparison with original version
B2A: Back translated version approved by instrument developers
CI: Cognitive interviewing
ICF: International Classification of Functioning, Disability and Health
IPM 1–4: The four levels of the Information Processing Model
IPM: Information Processing Model
ISPOR: International Society for Pharmacoeconomics and Outcomes Research
LS1-LS5: Life Space levels 1 to 5
LSA: Life Space Assessment
LSA-DK: Life Space Assessment in Danish
NMS: New Mobility Score
OMC: Orientation Memory Concentration test
QAS-99: Question Appraisal System
T1-T3: Forward translations by three independent translators
T4: Consensus forward translation
UAB: University of Alabama at Birmingham

## References

[1] Satariano WA, Guralnik JM, Jackson RJ, Marottoli RA, Phelan EA, Prohaska TR (2012). Mobility and aging: new directions for public health action. Am J Public Health.

[2] Studenski S, Perera S, Patel K, Rosano C, Faulkner K, Inzitari M (2011). Gait speed and survival in older adults. JAMA J Am Med Assoc.

[3] Brown CJ, Flood KL (2013). Mobility limitation in the older patient: a clinical review. JAMA J Am Med Assoc..

[4] Groessl EJ, Kaplan RM, Rejeski WJ, Katula JA, King AC, Frierson G (2007). Health-related quality of life in older adults at risk for disability. Am J Prev Med.

[5] Guralnik JM, Ferrucci L, Simonsick EM, Salive ME, Wallace RB (1995). Lower-extremity function in persons over the age of 70 years as a predictor of subsequent disability. N Engl J Med.

[6] Volpato S, Cavalieri M, Sioulis F, Guerra G, Maraldi C, Zuliani G (2011). Predictive value of the short physical performance battery following hospitalization in older patients. J Gerontol A Biol Sci Med Sci.

[7] Hirvensalo M, Rantanen T, Heikkinen E (2000). Mobility difficulties and physical activity as predictors of mortality and loss of independence in the community-living older population. J Am Geriatr Soc.

[8] Shumway-Cook A, Ciol MA, Yorkston KM, Hoffman JM, Chan L (2005). Mobility limitations in the Medicare population: prevalence and sociodemographic and clinical correlates. J Am Geriatr Soc.

[9] Culhane KM (2005). Accelerometers in rehabilitation medicine for older adults. Age Ageing.

[10] Pedersen MM, Bodilsen AC, Petersen J, Beyer N, Andersen O, Lawson-Smith L (2013). Twenty-four-hour mobility during acute hospitalization in older medical patients. J Gerontol A Biol Sci Med Sci.

[11] Bohannon RW (1995). Sit-to-stand test for measuring performance of lower extremity muscles. Percept Mot Skills.

[12] Aadahl M, Joergensen T (2003). Validation of a new self-report instrument for measuring physical activity. Med Sci Sports Exerc.

[13] Kristensen MT, Bandholm T, Holm B, Ekdahl C, Kehlet H (2009). Timed up & go test score in patients with hip fracture is related to the type of walking aid. Arch Phys Med Rehabil.

[14] Studenski S, Perera S, Wallace D, Chandler JM, Duncan PW, Rooney E (2003). Physical performance measures in the clinical setting. J Am Geriatr Soc.

[15] Peel NM, Kuys SS, Klein K (2013). Gait speed as a measure in geriatric assessment in clinical settings: a systematic review. J Gerontol A Biol Sci Med Sci.

[16] World Health Organization (WHO). Towards a Common Language for Functioning, Disability and Health: ICF, The International Classification of Functioning, Disability and Health. 2002.

[17] Baker PS, Bodner EV, Allman RM (2003). Measuring life-space mobility in community-dwelling older adults. J Am Geriatr Soc.

[18] Peel C, Sawyer Baker P, Roth DL, Brown CJ, Brodner EV, Allman RM (2005). Assessing mobility in older adults: the UAB study of aging life-space assessment. Phys Ther.

[19] Tsai L-T, Portegijs E, Rantakokko M, Viljanen A, Saajanaho M, Eronen J (2015). The association between objectively measured physical activity and life-space mobility among older people. Scand J Med Sci Sports.

[20] Auger C, Demers L, Gélinas I, Routhier F, Jutai J, Guérette C (2009). Development of a French-Canadian version of the life-space assessment (LSA-F): content validity, reliability and applicability for power mobility device users. Disabil Rehabil Assist Technol.

[21] Curcio C-L, Alvarado BE, Gomez F, Guerra R, Guralnik J, Zunzunegui MV (2013). Life-space assessment scale to assess mobility: validation in Latin American older women and men. Aging Clin Exp Res.

[22] Kammerlind A-SC, Fristedt S, Ernsth Bravell M, Fransson EI (2014). Test-retest reliability of the Swedish version of the life-space assessment questionnaire among community-dwelling older adults. Clin Rehabil.

[23] Fristedt S, Dahl AK, Wretstrand A, Björklund A, Falkmer T (2014). Changes in community mobility in older men and women. A 13-year prospective study. PLoS One.

[24] Ji M, Zhou Y, Liao J, Feng F (2015). Pilot study on the Chinese version of the life space assessment among community-dwelling elderly. Arch Gerontol Geriatr.

[25] Malterud K (2001). Qualitative research: standards, challenges, and guidelines. Lancet.

[26] Wild D, Grove A, Martin M, Eremenco S, McElroy S, Verjee-Lorenz A (2005). Principles of good practice for the translation and cultural adaptation process for patient-reported outcomes (PRO) measures: report of the ISPOR task force for translation and cultural adaptation. Value Health.

[27] Patrick DL, Burke LB, Gwaltney CJ, Leidy NK, Martin ML, Molsen E (2011). Content validity—establishing and reporting the evidence in newly developed patient-reported outcomes (PRO) instruments for medical product evaluation: ISPOR PRO good research practices task force report: part 2—assessing respondent understanding. Value Health.

[28] Willis GB (2005). Cognitive interviewing. A tool for improving questionnaire design.

[29] Drennan Jonathan (2003). Cognitive interviewing: verbal data in the design and pretesting of questionnaires. Journal of Advanced Nursing.

[30] Harkness J, Pennell B-A, Schoua-Glusberg A. Survey questionnaire translation and assessment. In: Presser S, Rothgeb JM, Couper MP, Lessler JT, Martin E, Martin J, Singer E (Eds.). Methods for testing and evaluating survey questionnaires. Hoboken, NJ: Wiley & Sons, Inc. p. 453–73.

[31] Thrasher JF, Quah ACK, Dominick G, Borland R, Driezen P, Awang R (2011). Using cognitive interviewing and behavioral coding to determine measurement equivalence across linguistic and cultural groups: an example from the international tobacco control policy evaluation project. Field Methods.

[32] Willis GB, Lessler JT (1999). Question Appraisal System QAS-99.

[33] Tourangeau R. Cognitive science and survey methods. In: Jabine T., Straf M., Tanur J. & Tourangeau R. cognitive aspects of survey methodology: building a bridge between disciplines. Washington: National Academic Press; 1984.

[34] Beaton D, Bombardier C, Guillemin F, Ferraz MB (2007). Recommendations for the cross-cultural adaptation of the DASH & QuickDASH outcome measures. Inst Work Health.

[35] Beatty PC, Willis GB (2007). Research synthesis: the practice of cognitive interviewing. Public Opin Q.

[36] Katzman R, Brown T, Fuld P, Peck A, Schechter R, Schimmel H (1983). Validation of a short orientation-memory-concentration test of cognitive impairment. Am J Psychiatry.

[37] Kristensen MT, Foss NB, Kehlet H (2005). Timed up and go and new mobility score as predictors of function six months after hip fracture. Ugeskr Laeger.

[38] Crabtree B, Miller W (1999). Doing qualitative research.

[39] Miles M, Huberman A, Salana J (2014). Qualitative data analysis - a methods sourcebook.

[40] COSMIN (COnsensus-based Standards for the selection of health Measurement INstruments) initiative. https://www.cosmin.nl/tools/cosmin-taxonomy-measurement-properties/. .

[41] Simões Maria do Socorro MP, Garcia Isabel FF, Costa Lucíola da CM, Lunardi Adriana C (2018). Life-Space Assessment questionnaire: Novel measurement properties for Brazilian community-dwelling older adults. Geriatrics & Gerontology International.

[42] Siordia C (2013). Evaluating response mechanisms in a life-space mobility instrument with a “stem and leaf” format. J Frailty Aging.

[43] Heesch KC, van Uffelen JG, Hill RL, Brown WJ (2010). What do IPAQ questions mean to older adults?. Lessons from cognitive interviews Int J Behav Nutr Phys Act.

[44] Burke DM, Light LL (1981). Memory and aging: the role of retrieval processes. Psychol Bull.

[45] Fried LP, Young Y, Rubin G, Bandeen-Roche K, WHAS II (2001). Collaborative research group. Self-reported preclinical disability identifies older women with early declines in performance and early disease J Clin Epidemiol.

[46] Fristedt S, Kammerlind A-S, Ernsth MB, Fransson EI. Concurrent validity of the Swedish version of the life-space assessment questionnaire. BMC Geriatr. 2016;16. 10.1186/s12877-016-0357-4. 10.1186/s12877-016-0357-4 PMC5100326 27821138

[47] Iglesias CP, Birks YF, Torgerson DJ (2001). Improving the measurement of quality of life in older people: the York SF-12. QJM Int J Med.

[48] Brown CJ, Roth DL, Allman RM, Sawyer P, Ritchie CS, Roseman JM (2009). Trajectories of life-space mobility after hospitalization. Ann Intern Med.

[49] Brown CJ, Kennedy RE, Lo AX, Williams CP, Sawyer P (2016). Impact of Emergency Department Visits and Hospitalization on Mobility Among Community-Dwelling Older Adults. Am J Med.

[50] Fathi R, Bacchetti P, Haan MN, Houston TK, Patel K, Ritchie CS (2017). Life-space assessment predicts hospital readmission in home-limited adults. J Am Geriatr Soc.

[51] Kennedy RE, Sawyer P, Williams CP, Lo AX, Ritchie CS, Roth DL (2017). Life-space mobility change predicts 6-month mortality. J Am Geriatr Soc.

[52] Lo AX, Brown CJ, Sawyer P, Kennedy RE, Allman RM (2014). Life-space mobility declines associated with incident falls and fractures. J Am Geriatr Soc.

[53] Portegijs E, Iwarsson S, Rantakokko M, Viljanen A, Rantanen T (2014). Life-space mobility assessment in older people in Finland; measurement properties in winter and spring. BMC Res Notes.

[54] Sheppard KD, Sawyer P, Ritchie CS, Allman RM, Brown CJ (2013). Life-space mobility predicts nursing home admission over 6 years. J Aging Health.

[55] Malterud K, Siersma VD, Guassora AD (2016). Sample size in qualitative interview studies: guided by information power. Qual Health Res.

[56] Beaton DE, Bombardier C, Guillemin F, Ferraz MB (2000). Guidelines for the process of cross-cultural adaptation of self-report measures. Spine..

[57] Blair J, Conrad F, Ackermann AC, Claxton G. The effect of sample size on cognitive interview findings. In: Proceedings of the American Statistical Association. Citeseer; 2006.

[58] Johannesen C, Davidsen M, Christensen A. [Older adults’ health and well-being - the profile of older adults 2019]. Copenhagen: The National Board of Health; 2019. Accessed 24 Jul 2019.

[59] Conrad FG, Blair J (2009). Sources of error in cognitive interviews. Public Opin Q..

[60] Wright AM, Holliday RE (2007). Interviewing cognitively impaired older adults: how useful is a cognitive interview?. Mem Hove Engl.

